# Documentation Completeness and Nurses’ Perceptions of a Novel Electronic App for Medical Resuscitation in the Emergency Room: Mixed Methods Approach

**DOI:** 10.2196/46744

**Published:** 2024-01-05

**Authors:** Kin Cheung, Chak Sum Yip

**Affiliations:** 1 School of Nursing The Hong Kong Polytechnic University Hong Kong China (Hong Kong); 2 Accident and Emergency Department Tuen Mun Hospital Hong Kong China (Hong Kong); 3 Quality & Safety Office The Hong Kong Children's Hospital Hong Kong China (Hong Kong)

**Keywords:** tablet computer, nursing documentation, paper resuscitation record, electronic resuscitation record, medical resuscitation, electronic medical record, documentation, resuscitation, electronic health record, nurses’ perception, traditional paper record, nurse

## Abstract

**Background:**

Complete documentation of critical care events in the accident and emergency department (AED) is essential. Due to the fast-paced and complex nature of resuscitation cases, missing data is a common issue during emergency situations.

**Objective:**

This study aimed to evaluate the impact of a tablet-based resuscitation record on documentation completeness during medical resuscitations and nurses’ perceptions of the use of the tablet app.

**Methods:**

A mixed methods approach was adopted. To collect quantitative data, randomized retrospective reviews of paper-based resuscitation records before implementation of the tablet (Pre-App Paper; n=176), paper-based resuscitation records after implementation of the tablet (Post-App Paper; n=176), and electronic tablet-based resuscitation records (Post-App Electronic; n=176) using a documentation completeness checklist were conducted. The checklist was validated by 4 experts in the emergency medicine field. The content validity index (CVI) was calculated using the scale CVI (S-CVI). The universal agreement S-CVI was 0.822, and the average S-CVI was 0.939. The checklist consisted of the following 5 domains: basic information, vital signs, procedures, investigations, and medications. To collect qualitative data, nurses’ perceptions of the app for electronic resuscitation documentation were obtained using individual interviews. Reporting of the qualitative data was guided by Consolidated Criteria for Reporting Qualitative Studies (COREQ) to enhance rigor.

**Results:**

A significantly higher documentation rate in all 5 domains (ie, basic information, vital signs, procedures, investigations, and medications) was present with Post-App Electronic than with Post-App Paper, but there were no significant differences in the 5 domains between Pre-App Paper and Post-App Paper. The qualitative analysis resulted in main categories of “advantages of tablet-based documentation of resuscitation records,” “challenges with tablet-based documentation of resuscitation records,” and “areas for improvement of tablet-based resuscitation records.”

**Conclusions:**

This study demonstrated that higher documentation completion rates are achieved with electronic tablet-based resuscitation records than with traditional paper records. During the transition period, the nurse documenters faced general problems with resuscitation documentation such as multitasking and unique challenges such as software updates and a need to familiarize themselves with the app’s layout. Automation should be considered during future app development to improve documentation and redistribute more time for patient care. Nurses should continue to provide feedback on the app’s usability and functionality during app refinement to ensure a successful transition and future development of electronic documentation records.

## Introduction

### Background

The completeness of documentation of critical care events in the accident and emergency department (AED) is essential for (1) the continuity of patient care, (2) medicolegal issues [1], (3) improving accessibility to critical information needed for research [2], and (4) serving as evidence for quality outcome measures [3]. Traditionally, documentation is performed on paper. Due to the fast-paced and complex nature of resuscitation cases, missing data is a common issue during emergency situations. As much as 60% of essential data fields in prehospital paper records can be incomplete [4]. A study in a trauma center also found incompleteness in 18% of the mandatory elements for trauma resuscitation [5].

Over the last 2 decades, there has been a global trend of switching to electronic medical records (EMRs). It was estimated that about 46% of AEDs in the United States used EMRs in 2010 [6]. This percentage is expected to increase in the future. Fully functional EMRs have been shown to improve efficiency in AEDs [7]. Despite the growth in usage, very few studies have explored the impact of EMRs in AED settings. Furthermore, the perceptions toward EMRs are mixed. A study found that nurses and physicians generally had a negative perception toward EMRs in the AED. EMRs are considered to be ineffective, redundant, and prone to error [8]. In contrast, another study found that nurses perceived that their productivity increased and care was better coordinated after implementing EMRs [9].

Among the limited studies in the area, a retrospective review of trauma resuscitations in AED settings showed that EMRs can improve documentation completeness [5,10]. However, there is a lack of studies on medical resuscitations, which are more common than trauma resuscitations [11]. Medical resuscitations are performed on triage category I and II patients with life-threatening conditions such as myocardial infarction, sepsis, and stroke [12]. Medical resuscitations differ from trauma resuscitations in that they do not follow a single protocol. The differences in management protocols can make the process of documenting medical resuscitations different from that of trauma resuscitations.

The EMR system used by the Hospital Authority of Hong Kong is called the Clinical Management System (CMS). It is an integrated platform that allows clinical users to manage the following daily clinical activities [13]: (1) obtain clinical data including consultation notes, laboratory, and imaging results; (2) document clinical activities; and (3) provide clinical decision support.

In AEDs in Hong Kong, medical records are still part paper and part electronic, with the patient’s clinical notes being documented on paper. This type of mixed documentation has been shown to hinder effective communication and utilization of information in either record [14].

Recently, there has been a trend of switching to electronic documentation in AEDs. In 2020, 3 of 18 AEDs in Hong Kong had switched to an EMR system called the eAED. It was expected that, by 2023, about two-thirds of AEDs would have switched to the eAED. The eAED is meant to replace paper charts previously used to document a patient’s progress [15]. Despite the gradual adoption of EMRs in AEDs in Hong Kong, the use of electronic documentation during medical resuscitations has not occurred owing to the time-critical, fast-paced nature and lack of a suitable application.

However, with advances in computer processing power, a tablet-based system could fill the gap. Documentation efficiency and data precision have improved when a tablet-based app was used, in comparison with a desktop EMR, during a simulation [16]. In Hong Kong, a tablet-based system called “eResus” is being developed by the Hong Kong Hospital Authority for medical and trauma resuscitation documentation. With the implementation of the eAED and eResus, documentation in AEDs would become fully electronic.

### Aim and Objectives

This study aimed to evaluate the impact of a tablet-based app on documentation completeness during medical resuscitations. The research questions were the following:

- What are the differences between paper and electronic tablet-based records on the levels of documentation completeness?
- What are the perceptions of emergency room nurses regarding documentation completeness when using eResus?

### Hypothesis

This study hypothesized that the completeness of resuscitation documentation using electronic tablet-based records would be higher than that that using paper records.

## Methods

### Design

To answer research question 1, a randomized retrospective review of paper and electronic resuscitation medical records (N=528) was conducted using a documentation completeness checklist. The study was implemented during the transition from paper to electronic documentation, when only triage category II cases would be documented using the tablet-based app called eResus. Therefore, triage category II records were collected before (from November 2020 to December 2020) and after (from February 2021 to March 2021) implementation of the tablet-based eResus app. Paper records were collected before (Pre-App Paper) and 1 month after (Post-App Paper) implementation of eResus, while electronic records were collected 1 month after (Post-App Electronic) implementation of eResus. We randomly selected 176 records each for the Pre-App Paper, Post-App Paper, and Post-App Electronic record sets from CMS using a random number generator.

To answer research question 2, emergency nurses’ perceptions of the advantages, challenges, and areas for improvement of the electronic app for resuscitation documentation were obtained in individual interviews conducted in mid-April 2021, 3 months after the implementation of eResus. Reporting of the qualitative findings was guided by the Consolidated Criteria for Reporting Qualitative Studies (COREQ) [17], as delineated in the following sections, to enhance rigor.

### Ethical Considerations

Ethical clearance (NTWC/REC/20098) from the study hospital and the Human Subjects Ethics Sub-committee of the Hong Kong Polytechnic University (HSEARS20200826001) was obtained before the commencement of the study.

### Data

#### Quantitative Data

A documentation completeness checklist was established based on the literature and a review of the department’s current medical resuscitation event documentation. The checklist consisted of 5 essential domains (ie, basic information, vital signs, procedures, investigations, and medications) of medical resuscitation as illustrated in Multimedia Appendix 1. Face and content validity of the checklist were determined by 4 experts in the emergency medicine field [18]. Experts were invited based on the following criteria: (1) worked in an AED and (2) published at least one article related to the accident and emergency field. The expert panel consisted of 1 associate consultant, 1 medical officer, and 2 advanced practice nurses (1 of which was a Fellow in Emergency Nursing). The content validity index (CVI) was calculated using the scale CVI (S-CVI). The S-CVI is calculated based on the number of items in the scale rated by the expert as “quite relevant” or “highly relevant” [19]. The S-CVI was further analyzed by universal agreement (UA) among experts (S-CVI/UA) and the average (S-CVI/Ave). The checklist’s S-CVI/UA was 0.822, and the S-CVI/Ave was 0.939.

The medical resuscitation documents were reviewed against the validated checklist by a researcher (CSY), and intrarater reliability was determined to ensure consistency. Intrarater agreement was calculated using the Cohen kappa [19]. We evaluated 5 cases at week 0 and week 2. The agreement between the 2 records was considered acceptable at a κ of 0.884 (95% CI 0.671-1.105; *P*<.001).

For each resuscitation documentation review, the researcher provided a dichotomous response of “Yes or No” for each item and identified the level of completeness based on the checklist. The patient diagnosis, length of medical resuscitation, initial triage category, and demographics including age and gender were also collected as part of the basic information. However, patient names and identification numbers were not collected. Data were kept anonymous by assigning codes only identifiable to the researcher.

#### Qualitative Data

Emergency room nurses were guided to discuss their thoughts on the eResus app’s features for documentation completeness through individual interviews with an onsite nurse who was one of the researchers (CSY). They understood the aim of the study, and their experience with the app was explored. Participants’ demographic data including age, gender, years of experience after graduation, and years of experience in their current specialty were collected for subsequent data analysis.

### Sample Size

For the quantitative data, the Chi-square test was used to compare the differences in documentation completeness between the 3 groups. Based on the findings from a level 1 pediatric trauma center in 2015 [10], with an α of .05 and power of 0.80, a minimum sample size of 153 medical records per record set was required. To ensure an adequate sample size, 176 patient records were included in each of the paper and electronic record sets, resulting in a total sample size of 528 records (ie, 176 records each for the Pre-App Paper, Post-App Paper, and Post-App Electronic record sets).

For qualitative data, data saturation is the criterion to determine the sample size. Data are considered saturated when no new theoretical insights are gained from new data [20]. For this study, data saturation was achieved after 10 individual interviews, and 2 more interviews were conducted to confirm the data saturation.

### Recruitment

The study was conducted in the AED of 1 hospital in Hong Kong. It is one the major local trauma centers providing 24-hour accident and emergency services and serves more than 190,000 patients per year, with over 300 resuscitation cases per month [21]. The tablet app was scheduled to be implemented in June 2020 but was postponed due to COVID-19. The app was eventually implemented in January 2021.

### Quantitative Data Collection Method

In this study, we reviewed 2 types of resuscitation documents, namely paper and tablet-based resuscitation records. Completed resuscitation documents in paper format were attached to the patient’s CMS record by optical scanning as per usual practice. These records were stored in the CMS.

Training prior to the implementation of the electronic resuscitation record could lead to bias toward improved completeness of electronic documents [10]. Therefore, paper documentation records were collected before and 1 month after implementation of eResus to address this issue. First, baseline paper resuscitation records were collected prior to implementation of the eResus app (Pre-App Paper). After the implementation of the eResus app with training, there was a washout period of 1 month. After 1 month, the paper (Post-App Paper) and tablet-based (Post-App Electronic) resuscitation records were retrieved for analysis. Both paper and tablet resuscitation records involving trauma team activation or triage category I cases were excluded because the app did not cover these 2 types of cases at this stage.

For the purpose of this study, 3 lists of 3 months of case lists including eligible medical records from the Pre-App Paper, Post-App Paper, and Post-App Electronic record sets were retrospectively generated from the CMS and assigned a serial number. From each group, 176 records were randomly selected using a random number generator.

### Qualitative Data Collection Method

In terms of qualitative data collection, purposive sampling was applied. The researcher conducted individual, voice-recorded interviews with each emergency nurse 3 months after the eResus implementation. The nurses were provided an explanation of the study, and written consent was obtained. The inclusion criteria included nurses (1) working in the AED of the hospital, (2) with experience using the eResus app, (3) who spoke Cantonese and were able to read English, and (4) working in their current position for more than 3 months.

Invitation emails were sent to colleagues. Eligible colleagues who replied to the email or expressed interest were invited to be interviewed according to their years of experience. Individual interviews were conducted in a quiet room or via Zoom. Each interview lasted about 1 hour or stopped when the interviewee felt that their viewpoint had been fully expressed. The interview guide is shown in Textbox 1.

**Interview guide.**

**Opening question:**

Can you tell me your experience with using the eResus app until now?

**Guiding questions:**

1. What are the main advantages and challenges with achieving high documentation completeness when using eResus in the resuscitation room?
2. How do you think eResus can be improved to help you achieve better documentation completeness?

### Data Analysis Methods

For the quantitative data analysis, SPSS version 25 (IBM Corp) was used. Descriptive statistics such as means, standard deviations, frequencies, and percentages were used to present the study variables. Normality was tested using the Kolmogorov-Smirnov test, and the data were found to be not normally distributed. The Mann Whitney *U* test was used to compare mean ranks for age and clinical characteristics between the Pre-App Paper and Post-App Paper record sets as well as between the Post-App Paper and Post-App Electronic record sets to ensure the clinical characteristics of the 3 groups were comparable. Subsequently, Chi-square tests were used to compare the differences in proportions, such as the percentage of completeness between the Pre-App Paper and Post-App Paper record sets to determine any historical bias or effect from training and then between the Post-App Paper and Post-App Electronic record sets. Results with a *P* value <.05 were considered significant.

Each resuscitation record was manually reviewed against the study checklist for data element completeness. Each record was reviewed individually. Any incomplete data element was entered as an incomplete domain for the respective domain of the 5 domains, namely basic information, vital signs, procedures, investigations, and medications. For example, for records of the administration of 2 medications that use the same route, the record was treated as 2 separate data entries. If 1 of the data items (such as 1 missing medication name) was incomplete, the medication domain for that case was entered as incomplete. The number of entries for each domain of the resuscitation documentation was analyzed, delineating sections that were recorded at higher or lower frequencies.

For the qualitative data analysis, content analysis was performed [22]. First, the interview was audio-recorded and transcribed verbatim into Chinese. NVivo Pro 12 was used for data analysis. The researcher read through the transcript multiple times to become immersed in the data. Participants’ experiences with the eResus app, challenges, and possible solutions were extracted and summarized into meaning units. Third, each meaning unit was condensed and labelled with codes. Fourth, subcategories were identified by comparing the similarities and differences between different codes. Finally, the latent meanings of the subcategories were sorted into themes.

## Results

### Quantitative Results

Tables 1 and 2 present the characteristics of the 5 domains of resuscitation documentation. Comparisons were made between the Pre-App Paper and Post-App Paper record sets. There were no significant differences in characteristics or documentation completion between the Pre-App Paper and Post-App Paper record sets (Table 3).

**Table 1:** Comparisons using the Mann-Whitney U test among the 3 resuscitation record sets in patient age; length of resuscitation; and total numbers of vital sign entries, procedures, investigations, and medications in the resuscitation documentation for patients requiring medical resuscitation (N=528).

5 domains			Pre-App Paper record set (n=176)					Post-App Paper record set (n=176)					Difference between Pre-App Paper and Post-App Paper record sets					Post-App Electronic record set (N=176)					Difference between Post-App Paper and Post-App Electronic record sets		
			Range		Mean (SD)		Range			Mean (SD)		*U*			*P* value		Range			Mean (SD)		*U*			*P* value
**Basic information**																									
	Age (years)	4-98		61.6 (20.6)		3-101			61.6 (22.0)		15,121			.84		4-100			62.5 (21.1)		15,188			.90	
	Length of resuscitation (minutes)	3-216		40.6 (30.2)		5-175			41.7 (28.6)		14,673			.39		8-367			40.5 (37.8)		14,214			.18	
Total number of vital sign entries			1-58		10.9 (8.0)		2-47			11.1 (7.1)		15,089			.68		2-72			9.9 (7.6)		13,258			.02
Total number of procedures			0-7		1.83 (1.34)		0-8			1.76 (1.26)		15,050			.61		0-10			1.50 (1.12)		13,900			.06
Total number of investigations			0-11		5.72 (1.89)		0-11			5.49 (1.92)		15,091			.67		0-11			5.70 (1.91)		14,741			.42
Total number of medications			0-12		1.65 (2.07)		0-15			1.70 (2.31)		15,085			.66		0-19			1.68 (2.41)		15,288			.83

**Table 2:** Gender differences among the 3 groups of resuscitation records for patients requiring medical resuscitation (N=528), as assessed using the Chi-square test.

Gender	Pre-App Paper record set (n=176), n (%)	Post-App Paper record set (n=176), n (%)	Post-App Electronic record set (n=176), n (%)	Difference among the groups	
				χ^2^ (df)	*P* value
Female	84 (47.7)	84 (47.7)	86 (48.9)	0.06 (2)	.97

**Table 3:** Differences in completion of the 5 domains of documentation between paper and electronic resuscitation records (N=528).

5 domains	Pre-App Paper record set (n=176), n (%)	Post-App Paper record set (n=176), n (%)	Post-App Electronic record set (n=176), n (%)	Differences among groups	
				χ^2^ (df)	*P* value
Basic information	113 (64.2)	105 (59.7)	128 (72.7)	6.86 (2)	<.001
Vital sign	116 (65.9)	108 (61.4)	158 (89.8)	40.97 (2)	<.001
Procedures	123 (69.9)	127 (72.2)	176 (100)	63.50 (2)	<.001
Investigations	101 (57.4)	93 (52.8)	128 (72.7)	16.06 (2)	<.001
Medications	158 (89.8)	163 (92.6)	175 (99.4)	15.24 (2)	<.001

For the post-app comparison, there were no significant differences in the characteristics, except the number of vital sign entries, between the Post-App Paper and Post-App Electronic record sets (Tables 1 and 2). To answer research question 1, there was a significantly higher completion rate for all 5 domains in the Post-App Electronic record set than in Post-App Paper record set (Table 3).

### Qualitative Results

#### Categories

The objective of the qualitative study was to explore nurses’ perceptions of the use of eResus for documentation completeness. The main categories identified were “advantages of tablet-based documentation of resuscitation records,” “challenges with tablet-based documentation of resuscitation records,” and “areas for improvement of tablet-based resuscitation records” (Textbox 2).

**Summary of the categories and subcategories.**

**Advantages of tablet-based documentation of resuscitation records**

- Structural guidance for documentation
- Easy to review and edit documentation
- Comparable mobility to paper and superior to desktop

**Challenges with tablet-based documentation of resuscitation records**

- System loading speed and stability
- Familiarization with the app layout

**Areas for improvement of tablet-based resuscitation records**

- Need for speedy documentation and automated documentation

Data saturation was achieved after conducting individual interviews with 12 nurses. The mean age of the participants was 26.9 (SD 2.68) years, and 9 participants were female. Participants’ mean length of work experience was 4.46 (SD 2.20) years, with a range of 2.5 years to 8.5 years. Their mean length of work experience in AED was 3.3 (SD 1.87) years, with a range of 1.5 years to 8.5 years.

#### Advantages of Tablet-Based Documentation of Resuscitation Records

##### Structural Guidance for Documentation

The electronic app included an extensive database that encompassed the essential aspects of resuscitation documentation. The participants appreciated the app’s preset data fields that prompted users to input essential data during documentation.

(During documentation of blood glucose,) the interface displayed all the data field such as time, result, performer. You definitely cannot forget to input.

D168-169

(After urinary catheter insertion) I may forget to write urinary output..., but eResus would prompt you if you did not enter.

H 170-173

The application made sure that you have 2 colleagues to countercheck the medication and documented their name before administration.

C164-165

The built-in logic set by emergency physicians and nurses provided clinical management support and guidance to users during documentation. Certain data fields were auto filled, saving more time for nursing care.

I found it convenient because the application would lead you how to input data in a step-by-step fashion.

A49

After inputting the systolic blood pressure, it would automatically divert you to the diastolic blood pressure.

C70-71

When asystole rhythm was chosen, the data field on blood pressure, pulse etc. would be prohibited from inputting...We no longer have to write “undetectable” over and over again.

E47-52

Furthermore, the electronic app reduced the need for verbal order prescriptions and allowed structured electronic prescriptions, which are less prone to error during documentation and administration.

Verbal order was prone to miscommunication, distraction, and error in administration.

C146-147

In the past, I would have to remember or write down physician’s verbal order..., but now the drug name, dosage, infusion speed etc. would all be on the screen.

K 41-47

##### Easy to Review and Edit Documentation

Medical resuscitation documentation has to be done contemporaneously during resuscitation. Electronic documentation can ensure legibility compared with handwriting, and users were able to review specific aspects of the documentation for completeness using in-app features.

Colleagues’ handwriting could be illegible; maybe everyone was in a hurry. And colleagues could misspell words, which could affect handover to ward colleagues.

E22-24

Someone may accidently splash alcohol onto the paper chart, and the word would become illegible.

H209-210

The application has a filter function which allows you to choose vital signs, allowing you to review vital sign inputs and trends or procedures, allowing you to review whether you have forgotten to document something.

K123-125

Fragmented information was conveyed to the documenter from various sources, in a random sequence. Not all users can correctly recall the exact sequence of medical resuscitation as they document. Electronic documentation allowed the users time to edit the sequence rather than having to rewrite the whole resuscitation event on a new paper resuscitation record.

If the handwriting was too ugly and the time sequence is too out of place, such as the medication administration time did not align to the corresponding row, then I would cross out the whole paper chart and rewrite it. But now, eResus can easily amend it.

I 104-106

##### Comparable Mobility to Paper and Superior to Desktop

Patients requiring medical resuscitation would often need to be transferred to another department for investigation or intervention. A tablet app can provide the mobility needed to document in various locations.

Let’s say the patient has to be escorted to computed tomography (CT). I would take the table to the CT suite (to continue the document). When we returned to the resuscitation bay, I could use the Bluetooth keyboard to continue the document. It’s better than desktop.

L 169-171

You can bring it (to CT) like paper...don’t even need to bring pen, just use your fingers.

J 179-180

#### Challenges With Tablet-Based Documentation of Resuscitation Records

##### System Loading Speed and Stability

Participants embraced the transition to electronic documentation. However, participants reported technical challenges due to the internet connection or app coding issues when using the electronic app that could compromise documentation completeness. The fast pace of medical resuscitations and contemporaneous nature of the documentation exacerbated the problem.

It has some technical problems...there was a time when it kept crashing and could not input data.

A58-60

Sometimes, switching between different tab pages is rather slow.

F60

The patient was...in asystole, we were conducting chest compression, and administering medication, but the application was still loading.

I 116-117

##### Familiarization With the App Layout

Navigating through the various tab bars, interface, and data fields of the app was different from the paper resuscitation record that presented all the data fields on the same page. Users were required to tab multiple times to access the desired data fields on the tablet, which was more time-consuming. All participants received training prior to using the electronic app in clinical settings. They believed that being familiar with the design of the app takes time and practice:

I have to tab this and that before I could input data...if it was handwritten, it would be much quicker.

H 53-57

This application has different tabs and options, which require a bit of thinking...it is like using a phone.

C33-43

When you first encounter the application, you would need to spend time to learn the layout. But after you have become familiar with it, you would find the documentation process very smooth.

D147-149

#### Areas for Improvement of Tablet-Based Resuscitation Records

All participants reported that speed of documentation was an important aspect in resuscitation documentation. During medical resuscitations, the case nurse was required to perform patient care and document contemporaneously. These resuscitation events were highly demanding and required speedy documentation:

Sometime, the documentation with eResus could take up lots of time. There were cases when we needed to document lots of medication right at the beginning. The application may not be able to document events in real time.

A63-64

When you have many items pending documentation, you would be naturally prone to incomplete documentation.

B76-77

The multitasking nature of the nursing practice posed competing demands between managing patient care and documentation, which required the nurses to compromise. Nurses decreased the frequency of taking vital signs. One participant said:

When handling less critical cases...I would take vital signs every 5 minutes (instead of 3) so that I can be more at ease when managing both the patient and documentation.

D113-117

Users appreciated the auto retrieval of data from the Hospital Authority’s network and the auto fill of relevant fields. Relevant data previously inputted into the app were prepopulated either automatically or after the user’s approval:

It would auto-capture allergy status from CMS, an electronic health record system used in Hong Kong).

G 102-103

It can retrieve the previous (vital sign) data. Then, I can tab it and paste it onto the data field...such as Glasgow Coma Scale score, etc.

H 44-46

Faster documentation speed can improve documentation completeness and overall resuscitation quality. Participants believed that speed and improved care could be gained by automation:

If a multimonitor could automatically record vital signs and transfer data into the application, the user would spend less time inputting data and more time looking after the patient...or checking the (resuscitation) record for incompletion.

D254-262

(Automated vital sign recording) would be useful...But some factors could affect the reading’s accuracy; it should allow health care workers to verify the readings prior to documenting.

K 217-220

Documentation speed can also be gained by flattening the user interface (UI). Participants found that, although organizing data fields into different categories and layers was logical, it made navigating through the layers inevitably slow. A more direct, intuitive UI is needed to improve the speed of data entry:

(The commonly used) items should be accessible with one tap.

G194

The commonly performed investigations...that has many data fields should be more easily accessible.

L 216-220

## Discussion

### Principal Findings

This is the first study, particularly in Asia, to compare the completion rates of documentation between paper and tablet-based resuscitation records in the emergency room. Our results indicate that electronic documentation is promising, with a higher completion rate than with the paper format.

Our study results support the hypothesis that tablet-based documentation of resuscitation records results in a higher documentation completion rate than paper formats in all 5 domains. Previous studies in AED settings have been conducted to compare the completion rates of key data elements for trauma resuscitation records between paper and electronic formats for adult [23] and pediatric [10] trauma cases. Both studies found areas for improvement and degradation in the key data elements. However, it is difficult to directly compare the studies since the outcome measures were different and none of the studies in AED settings used tablet-based devices. Nevertheless, our study results were consistent with those of previous studies that supported that, with electronic resuscitation records, documentation completion rates were higher, particularly of basic information such as case start time and disposal but not for serial vital signs [10,23]. Interestingly, although no difference was found in the completeness of documenting vital signs and interventions in these previous studies, our study showed improvement in the vital signs, procedures, investigations, and medication domains. This may be due to the differences in the inherent design of the EMRs and the use of a tablet-based device instead of desktops, as explained by interview participants. In addition, contrary to the concern of bias for improved documentation completeness caused by training [10], our study showed no statistically significant differences between the Pre-App Paper and Post-App Paper record sets. This indicates that missing data with the paper format could be consistent since the documentation format has not changed (such as using the same paper form).

Our qualitative results further explain the reasons why the tablet-based device could improve documentation completeness. The structural design of the tablet-based resuscitation record provided guidance that contributed to the completeness. This guidance provides support to the documenter via various clinical support features such as structured prescriptions, preset data fields, and preset documentation logic. This structural guidance was developed by consulting local emergency physicians and nurses working in the AED. The guidance mimicked the normal workflow and thus supported the documentation process. Similar results were found in a previous study in which nurses had higher confidence using the EMR when they perceived that their suggestions were used to customize the system [24]. This also implies that the tablet-based device will be considered useful if it is country, institution, and department-specific.

However, similar to other studies, our qualitative findings supported that nurses have to multitask during work, which has been shown to compromise documentation completeness [8]. Furthermore, documentation in an EMR was perceived to be more time-consuming and complex [25]. Our study participants also experienced similar concerns with slow app loading speeds and needing to navigate through various tabbed pages, which increases the complexity of documentation.

Our participants further suggested that future development of the app should include automation features that would spare the documenter from manually inputting individual data into the app. Automating data input can reduce the documenters’ need to tab multiple times before finding the desired data field. This would be particularly useful for vital signs, which were the most frequently documented in this study. The UI should be flattened to facilitate input of other common data fields, which supports the concept that data fields that are more frequently recorded should be located in readily accessible spots [26]. With the automation of vital signs and an improved UI, the documenter would be able to spend more time on patient care and document in real time.

### Implications for Emergency Room Nurses

Most emergency room nurses believed that the transition from paper to electronic charting can improve the quality of resuscitation documentation and patient safety. This study clearly demonstrates the potential of electronic charting to achieve that. During the transition period, the nurse documenters faced general problems with resuscitation documentation such as multitasking and unique challenges such as software updates and a subsequent need to become familiar with the app’s layout. Therefore, systematic, periodic needs assessments of nurse documenters using tablet-based devices, followed by corresponding training, should be conducted. Emergency room nurses should also be actively involved in the development and implementation phases to ensure success in the transition and future development of electronic documentation. Automation functions should be considered during the development of future apps to improve documentation and redistribute more time for patient care.

### Limitations

This study has some limitations. It was not able to demonstrate the effect on documentation accuracy, rates of medication errors, the quality of patient care, or the process of clinical decision-making. Furthermore, since the sample was obtained from 1 AED only, the study findings may not be generalizable to other AEDs or other acute ward settings where the staffing and workflow may be different. In addition, this study excluded trauma cases and cardiopulmonary resuscitation cases; thus, its findings cannot be generalized to all resuscitation room situations in the AED.

### Conclusions

This study demonstrates that a statistically higher completion rate in 5 domains essential to resuscitation documentation was achieved with a tablet-based device than with the traditional paper resuscitation documentation. Refinement of the device should be ongoing and include consultation with the users. Further studies can expand the scope to involve all medical resuscitation cases across AEDs.

## Appendix

Multimedia Appendix 1 Documentation completeness checklist.

## Abbreviations

AED: accident and emergency department
CMS: Clinical Management System
COREQ: Consolidated Criteria for Reporting Qualitative Studies
CT: computed tomography
CVI: content validity index
EMR: electronic medical record
S-CVI: scale content validity index
UA: universal agreement
UI: user interface
