# Supplementary material for: Documentation Completeness and Nurses’ Perceptions of a Novel Electronic App for Medical Resuscitation in the Emergency Room: Mixed Methods Approach
Source: JMIR Mhealth Uhealth. 2024 Jan 5;12:e46744. doi: 10.2196/46744 (PMC10799286; doi:10.2196/46744)
Supplement: Multimedia Appendix 1 [file mhealth_v12i1e46744_app1.pdf]

Documentation completeness checklist

Domain 1: Basic information and miscellaneous

Definition: Documentation of relevant case information during resuscitations. Each parameter may consist of various sub-items.

Item	Sub-item	Complete
Drug allergy status	Specify yes/no/ unknown	Yes☐ No☐
Case start time		Yes☐ No☐
Case disposal time		Yes☐ No☐
Consultation	Call time	Yes☐ No☐
	Respond time	Yes☐ No☐
Name of case nurse		Yes☐ No☐
Name of case MO		Yes☐ No☐

Domain 2: Vial sign

Definition: Documentation of physiological parameters of patients during resuscitations. Each parameter may consist of various sub-items.

Item	Sub-item	Complete
Time should be documented in ALL items		Yes☐ No☐
Cardiac Rhythm (Rhythm)	Name of rhythm	Yes☐ No☐
Blood Pressure (BP)	SBP (mmHg)	Yes☐ No☐
	DBP (mmHg)	Yes☐ No☐
	If SBP or DBP not a/v, remarks such as undetectable, carotid pulse present should be recorded.	Yes☐ No☐
Pulse rate (P)	Number of beats (/min)	Yes☐ No☐
	If no pulse, should record undetectable	Yes☐ No☐
Respiratory rate (RR)	Number of breaths (/min)	Yes☐ No☐
O2 Adjunct	Name of O2 adjunct	Yes☐ No☐
	Litre of O2 (L)	Yes☐ No☐
Saturation of Peripheral Oxygen (SpO2)	Number (%)	Yes☐ No☐
Glasgow Coma Scale	Number or alphabet	Yes☐ No☐

(GCS)		
Pupil	Size	Yes☐ No☐
	Reactivity	Yes☐ No☐
Temperature (Temp)	Number (°C)	Yes☐ No☐
End Tidal CO2 (ETCO2)	Number (mmHg)	Yes☐ No☐

Domain 3: Procedure

Definition: Documentation of procedures performed to patients during resuscitations. Each parameter may consist of various sub-items.

Item	Sub-item	Complete
IV line	Site	Yes☐ No☐
IO line	Site	Yes☐ No☐
C-Collar	Collar application time	Yes☐ No☐
	Collar off time	Yes☐ No☐
Spinal board	On time	Yes☐ No☐
	Off time	Yes☐ No☐
Splints	On time	Yes☐ No☐
	Off time	Yes☐ No☐
	Site	Yes☐ No☐
	Type	Yes☐ No☐
	Post splint circulation	Yes☐ No☐
Pelvic binder	On time	Yes☐ No☐
	Off time	Yes☐ No☐
Hemostatic tourniquet	On time	Yes☐ No☐
	Off time	Yes☐ No☐
	Site	Yes☐ No☐
	Adequacy of bleeding control	Yes☐ No☐
Time should be documented in the following items		Yes☐ No☐
Suture of wound	Site	Yes☐ No☐
Chest seal	Location	Yes☐ No☐
CPR	Start time	Yes☐ No☐
	Stop time	Yes☐ No☐
	Choice of mechanical CPR device (e.g. Manual, LUCAS)	Yes☐ No☐

Defibrillation	Number of Joule	Yes☐ No☐
	Mode: mono vs biphasic	Yes☐ No☐
	Pad location: antero-apical vs antero-posterior	Yes☐ No☐
Cardioversion	Number of Joule	Yes☐ No☐
	Mode: mono vs biphasic	Yes☐ No☐
	Pad location: antero-apical vs antero-posterior	Yes☐ No☐
	Shaving or not	Yes☐ No☐
	Sedation	Yes☐ No☐
Transcutaneous pacing	Current dosage (mA)	Yes☐ No☐
	Pacing rate: pulse/min	Yes☐ No☐
	Sedation	Yes☐ No☐
	Mode: (demand vs mandatory	Yes☐ No☐
Blood product transfusion	Type of blood product	Yes☐ No☐
	Matching: matched vs unmatched	Yes☐ No☐
	Infusion line location	Yes☐ No☐
	Volume infused	Yes☐ No☐
	Infusion rate	Yes☐ No☐
	MTG batch no	Yes☐ No☐
	Prescribed by	Yes☐ No☐
	Admin by	Yes☐ No☐
	Checked by	Yes☐ No☐
	Specific blood product detail: e.g. CMV neg, irradiated	Yes☐ No☐
ECMO	Type: VA, VV	Yes☐ No☐
IO line	Site	Yes☐ No☐
Central line insertion	Site	Yes☐ No☐
	Type of catheter	Yes☐ No☐
A-line	Site	Yes☐ No☐
	Size (G)	Yes☐ No☐
Blood Loss	Type: e.g. wound/ vaginal/ drain	Yes☐ No☐
	Volume of blood loss	Yes☐ No☐

ETT cuff pressure	Number: pressure	Yes☐ No☐
Needle Thoracostomy	Site	Yes☐ No☐
	Volume of output	Yes☐ No☐
	Nature of output	Yes☐ No☐
Chest Drainage	Site	Yes☐ No☐
	Tube size (Fr)	Yes☐ No☐
	Local anesthetics type	Yes☐ No☐
	Local anesthetics volume	Yes☐ No☐
	Nature of output	Yes☐ No☐
	Presence of swinging	Yes☐ No☐
	Volume of output	Yes☐ No☐
	Tube marking	Yes☐ No☐
Pericardiocentesis	Method: e.g. Subxiphoid, precordial	Yes☐ No☐
	Volume of output	Yes☐ No☐
	Nature of output	Yes☐ No☐
Gastric-Tube	Site of tube	Yes☐ No☐
	Tube size (Fr)	Yes☐ No☐
	Nature of output	Yes☐ No☐
	Volume of output	Yes☐ No☐
	To BSB or not	Yes☐ No☐
Gastric lavage	Method (open or closed)	Yes☐ No☐
	Volume of input	Yes☐ No☐
	Volume of output	Yes☐ No☐
	Nature of output	Yes☐ No☐
	Post lavage activated charcoal?	Yes☐ No☐
Urinary Catheter	Type	Yes☐ No☐
	Size (Fr)	Yes☐ No☐
	Output (ml)	Yes☐ No☐
	Nature of urine	Yes☐ No☐
Rewarming	Type (e.g. Bair Hugger, ReadyHeat)	Yes☐ No☐
External cooling	Type (e.g. blanketrol)	Yes☐ No☐
Physical restraint	type	Yes☐ No☐
	Site	Yes☐ No☐
Co-oximetry	Carboxyhemoglobin (%)	Yes☐ No☐

	Methemoglobinm(%)	Yes☐ No☐
Bladder irrigation	Volume of input	Yes☐ No☐
	Volume of output	Yes☐ No☐
	Nature of fluid	Yes☐ No☐
Doptone	Heart beat per min	Yes☐ No☐
Baby delivery	Delivery location	Yes☐ No☐
	Delivery method	Yes☐ No☐
	Position (e.g. breech, cephalic)	Yes☐ No☐
	Episiotomy (yes or no)	Yes☐ No☐
	Perineal tear (e.g. degree of tear)	Yes☐ No☐
	Blood loss	Yes☐ No☐
	Aseptic/ septic cut cord	Yes☐ No☐
Placenta delivery	Completeness of placenta	Yes☐ No☐
	Weight (g)	Yes☐ No☐
	Blood loss (ml)	Yes☐ No☐
	Cord blood taken or not	Yes☐ No☐
Flexible laryngoscopy	Start time	Yes☐ No☐
	End time	Yes☐ No☐
		Yes☐ No☐
Oxylog 1000 - CMV	Pmax (mbar/cmH2O)	Yes☐ No☐
	Freq./RR (/min)	Yes☐ No☐
	MV (L/min)	Yes☐ No☐
	FiO2	Yes☐ No☐
Oxylog 3000 - IPPV	Pmax (mbar/cmH2O)	Yes☐ No☐
	Freq./RR (/min)	Yes☐ No☐
	VT /TV (mL)	Yes☐ No☐
	FiO2 (%)	Yes☐ No☐
	PEEP (mbar/cmH2O)	Yes☐ No☐
	Trigger (L/min)	Yes☐ No☐
	Ventilation time ratio (I:E)	Yes☐ No☐
	Tplat (%)	Yes☐ No☐
Oxylog 3000 - SIMV	Pmax (mbar/cmH2O)	Yes☐ No☐
	Freq./RR (/min)	Yes☐ No☐
	VT /TV (mL)	Yes☐ No☐
	FiO2 (%)	Yes☐ No☐

	PEEP (mbar/cmH2O)	Yes☐ No☐
	Trigger (L/min)	Yes☐ No☐
	Tplat (%)	Yes☐ No☐
	△ Psupp/ ASB (mbar/cmH2O)	Yes☐ No☐
	Tinsp (s)	Yes☐ No☐
Oxylog 3000 - BIPAP	Pmax (mbar/cmH2O)	Yes☐ No☐
	Freq./RR (/min)	Yes☐ No☐
	FiO2 (%)	Yes☐ No☐
	PEEP (mbar/cmH2O)	Yes☐ No☐
	Trigger (L/min)	Yes☐ No☐
	△ Psupp/ ASB (mbar/cmH2O)	Yes☐ No☐
	Pinsp (mbar/cmH2O)	Yes☐ No☐
	Tinsp (s)	Yes☐ No☐
Oxylog 3000 - CPAP	Pmax (mbar/cmH2O)	Yes☐ No☐
	FiO2 (%)	Yes☐ No☐
	PEEP (mbar/cmH2O)	Yes☐ No☐
	Trigger (L/min)	Yes☐ No☐
	△ Psupp/ ASB (mbar/cmH2O)	Yes☐ No☐
OP airway	Size	Yes☐ No☐
NP airway	Size	Yes☐ No☐
LMA	Size	Yes☐ No☐
ETT	Tube size	Yes☐ No☐
	Marking (cm)	Yes☐ No☐
	Cuff pressure (cm H2O)	Yes☐ No☐
NTT	Tube size	Yes☐ No☐
	Marking (cm)	Yes☐ No☐
	Cuff pressure (cm H2O)	Yes☐ No☐
	ETT type	Yes☐ No☐
Needle Cricothyroidotomy	Size (G)	Yes☐ No☐
Surgical Cricothyroidotomy	Tube type (ETT vs tracheostomy tube)	Yes☐ No☐
	Cuffed/non-cuffed	Yes☐ No☐
	Size	Yes☐ No☐

Tracheostomy	Tube size	Yes☐ No☐
	Method (open vs percutaneous)	Yes☐ No☐
	Cuffed/ non-cuffed	Yes☐ No☐
Removal of airway foreign body	Type of FB	Yes☐ No☐
	Location of FB	Yes☐ No☐
O2	Name of O2 adjunct	Yes☐ No☐
	Flow rate	Yes☐ No☐
Suction		Yes☐ No☐
	Nature of output	Yes☐ No☐
	Volume of output	Yes☐ No☐
Assisted ventilation (BVM)		Yes☐ No☐

Domain 4: Investigations

Definition: Documentation of investigations performed to patients during resuscitations. Each parameter may consist of various sub-items.

Item	Sub-item	Complete
Time should be documented in ALL items		Yes☐ No☐
POCT Hb	Result (mmol/L)	Yes☐ No☐
POCT Blood Glucose	Result (mmol/L)	Yes☐ No☐
POCT Blood gas + electrolyte	Blood sample type (capillary/ venous/ artery)	Yes☐ No☐
POCT Lactate	Result (mmol/L)	Yes☐ No☐
ROTEM		Yes☐ No☐
Multistix	Result	Yes☐ No☐
POCT urine pregnancy test	Result	Yes☐ No☐
urine toxi	Result	Yes☐ No☐
ECG	Text: type e.g. right/ left/ posterior	Yes☐ No☐
X-Ray	Region e.g. chest, AXR	Yes☐ No☐
CT	Region e.g. chest, AXR	Yes☐ No☐
USG	Type of exam: FAST, IVC	Yes☐ No☐

Domain 5: Medication

Definition: Documentation of medication during resuscitations. Each parameter may consist of various sub-items.

Item	Sub-item	Complete
Oral, sublingual, Inhalation medication	Prescriber	Yes☐ No☐
	Drug name	Yes☐ No☐
	Dosage	Yes☐ No☐
	Route	Yes☐ No☐
	Administration time	Yes☐ No☐
	Admin by	Yes☐ No☐
Subcutaneous IM, IV bolus, slow IV, IV intermittent, IV continuous medication	Prescriber	Yes☐ No☐
	Drug name	Yes☐ No☐
	Dosage	Yes☐ No☐
	Route	Yes☐ No☐
	Dilution method if appropriate	Yes☐ No☐
	Administration time	Yes☐ No☐
	Admin by	Yes☐ No☐
	Check by	Yes☐ No☐
	Site of IV line used	Yes☐ No☐
IO medication	Prescriber	Yes☐ No☐
	Drug name	Yes☐ No☐
	Dosage	Yes☐ No☐
	Route	Yes☐ No☐
	Dilution method if appropriate	Yes☐ No☐
	Administration time	Yes☐ No☐
	Admin by	Yes☐ No☐
	Check by	Yes☐ No☐
	Site of IO line used	Yes☐ No☐
